# Entrapment of the Superficial Peroneal Nerve at a Band Proximal to the Crural Fascia: A Cadaveric Case Report

**DOI:** 10.7759/cureus.83734

**Published:** 2025-05-08

**Authors:** Christos Lyrtzis, Christos Tsiantas, Apostolos Prinos, Maria Tzika, George Paraskevas

**Affiliations:** 1 Department of Anatomy and Surgical Anatomy, Aristotle University of Thessaloniki, Thessaloniki, GRC; 2 School of Medicine, Aristotle University of Thessaloniki, Thessaloniki, GRC; 3 Department of Anatomy and Surgical Anatomy, Faculty of Health Sciences, Medical School, Aristotle University of Thessaloniki, Thessaloniki, GRC

**Keywords:** bundle, cadaver, entrapment, peroneal nerve, superficial

## Abstract

The superficial peroneal nerve (SPN), a branch of the common peroneal nerve, typically courses through the anterolateral compartment of the leg and emerges by piercing the crural fascia in the distal third of the leg. Although rare, entrapment of the SPN may occur at its fascial exit point and can lead to sensory symptoms in its cutaneous distribution. According to existing literature, entrapment sites are usually located 50-100 mm proximal to the lateral malleolus. In this dissection report, we present a case of SPN entrapment in a 75-year-old male, observed 47 mm proximal to the lateral malleolus, where the nerve was compressed beneath a fibrous band at its point of emergence through the crural fascia. The precise location of SPN compression is clinically significant for the accurate diagnosis of entrapment syndromes and for guiding surgical intervention. Awareness of such topographical variants is essential in the evaluation and management of chronic lower leg pain.

## Introduction

The superficial peroneal nerve (SPN), also known as the superficial fibular nerve (SFN), is a branch of the common peroneal nerve, which arises from the L4, L5, S1, and S2 spinal roots. This nerve demonstrates standard bifurcation as it wraps around the fibular neck, passing inferior to the peroneus longus muscle [1]. Following its bifurcation, the SPN typically traverses the lateral compartment of the leg, descending obliquely between the peroneus longus, peroneus brevis, and extensor digitorum longus muscles, to which it provides motor innervation in close association with the anterior intermuscular septum [2]. At the lower third of the leg, SPN is found below the crural fascia, while it gradually shifts to a more superficial plane before eventually piercing it approximately 10 cm proximally to the lateral malleolus. After that, it provides sensory innervation to the leg's anterolateral surface, superior aspect of the foot, and all the dorsal part of the toes, except the 1st interdigital space, by dividing into its final branches the intermediate and medial dorsal cutaneous nerves [3].

Entrapment of the SPN has been reported in the literature, causing inversion injuries of the ankle, severe pain, and Tinel's sign along the cutaneous branches of the SPN on the dorsum of the ankle and foot [4]. In a study by Styf and Morberg involving 480 patients with chronic leg pain, only 17 cases (3.5%) were diagnosed with SPN entrapment [5]. Similarly, Falciglia et al. assessed 921 pediatric patients presenting with acute ankle sprains and identified SPN entrapment in only 5 cases, suggesting that SPN entrapment is a relatively uncommon clinical entity in both chronic and acute lower limb conditions [6].

Thus, this study aims to present a cadaveric case of entrapment of the SPN by a fibrous band within the crural fascia near the lateral malleolus and provide a short review of the literature concerning this rare condition.

## Case presentation

**Figure 1:**
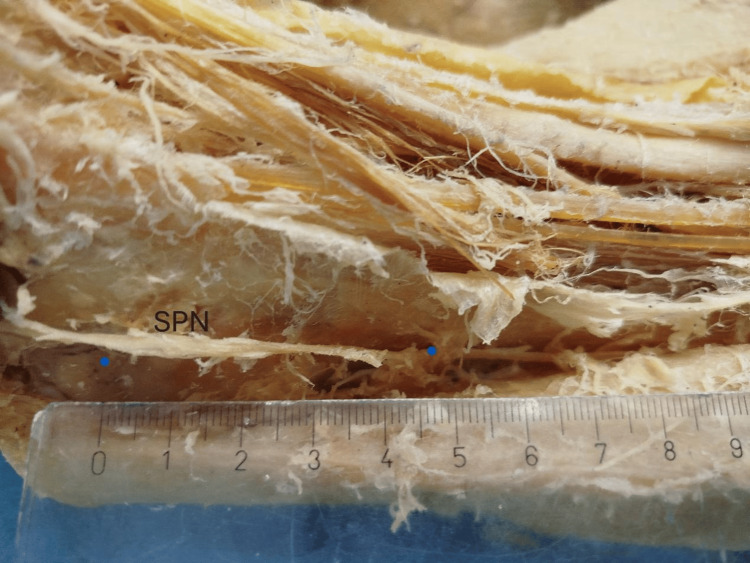
The distance between the location of the entrapment of the superficial peroneal nerve (SPN) and the lateral malleolus (blue dots) is 47 mm.

**Figure 2:**
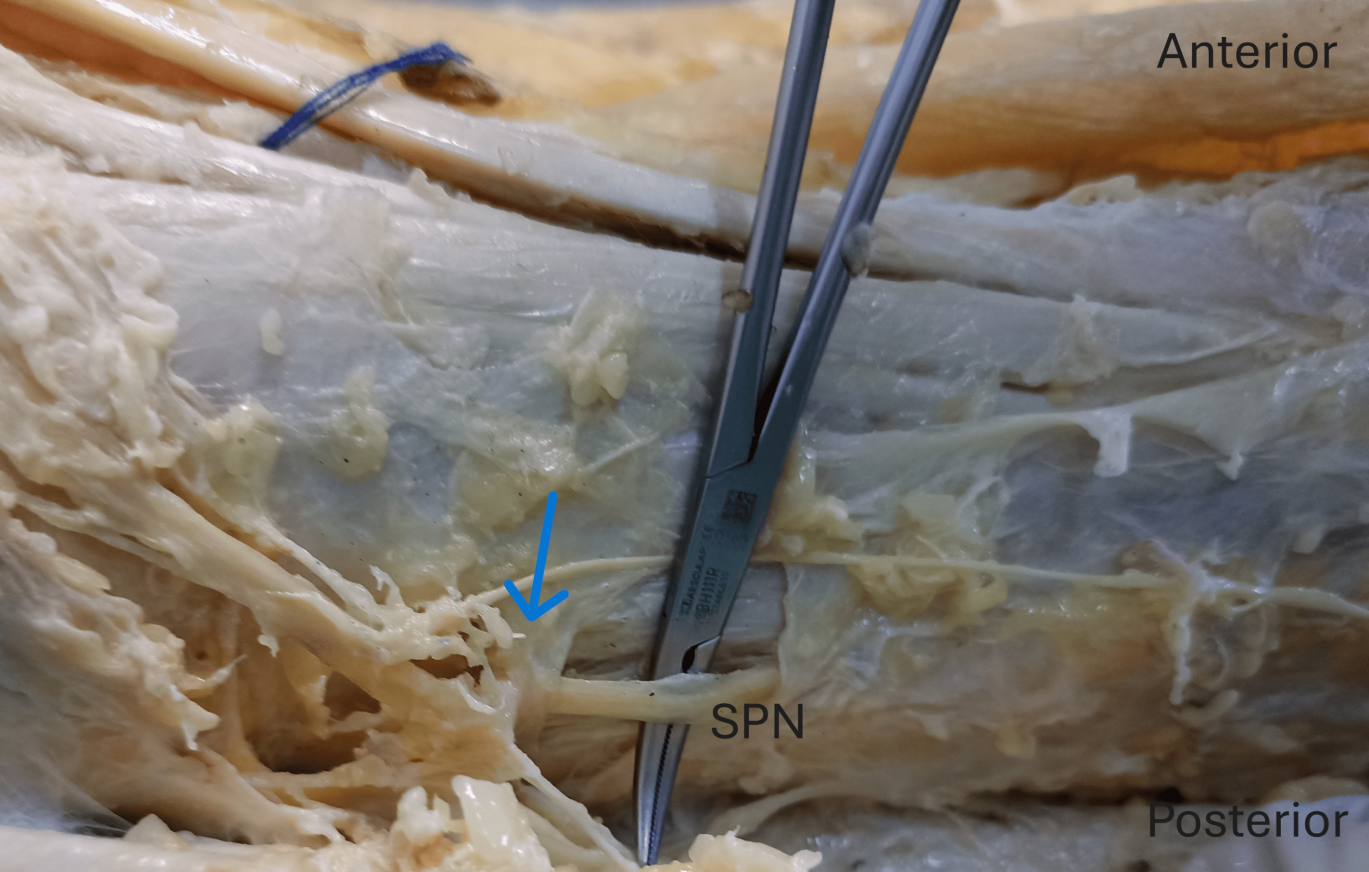
Fasciotomy of the anterior and lateral compartments of the left leg. The band (blue arrow) possibly entraps the superficial peroneal nerve (SPN).

During a course at the Anatomy Lab of the Faculty of Medicine, Aristotle University of Thessaloniki, we dissected a 75-year-old male cadaver with an unknown cause of death. The cadaver was positioned supine for the dissection. An incision was made along the anterior and lateral compartments of the left lower leg to expose the SPN. After the initial skin incision, the subcutaneous tissue was carefully dissected to expose the deeper muscles and fascial layers, ensuring that the superficial nerves and blood vessels were not damaged. This allowed for the identification of the SPN and a thorough examination of its course. Particular attention was paid to the nerve's relationship with adjacent muscles, fascial structures, and bony landmarks, such as the lateral malleolus, which serves as a key reference point for the nerve’s path. An unexpected morphological variation was noted during the dissection. A fibrous fascia was found possibly compressing the SPN at the location where it emerges beneath the crescent-shaped crural fascia (Figure 1). This compressive band was observed to be 5 mm in width, with its entrapment site positioned 47 mm proximal to the lateral malleolus (Figure 2). The possible compression was clearly affecting the nerve’s path and may potentially contribute to neuropathy or related clinical conditions. No additional anomalies or variations were noted in the lower extremity anatomy during the dissection.

## Discussion

Nerve entrapment syndrome is considered a pathological condition where there is compression of a nerve as it penetrates anatomic structures, such as a fibro-osseous tunnel or fascial passage [4]. According to research of 480 patients with chronic leg pain, only 17 patients were diagnosed with SPN entrapment [5]. The loss of mobility and flexibility of a peripheral nerve as it gets compressed by its surrounding tissues might cause motor or sensory deficits in the area of nervous distribution. SPN injury may arise secondary to mechanical stressors and space-occupying lesions. Etiological factors include repetitive stretch and inversion mechanisms, recurrent ankle sprains, and compressive soft tissue masses such as ganglia. External compression neuropathies have also been attributed to constrictive footwear, with fibrosis observed at the strap contact site. Furthermore, SPN compromise may occur due to direct trauma in the distal leg or indirectly following surgical intervention, such as hallux valgus correction. A rare instance of entrapment within fibular fracture callus has also been documented, highlighting potential osseous causes of nerve compression [7].

Entrapment of the SPN is rarely reported, and symptoms may include tingling and abnormal sensation localized to the lateral side of the lower leg and dorsum of the foot, with the first web space remaining unaffected. Pain is typically aggravated by physical exertion. Deep palpation of the nerve may not always lead to positive Tinel’s sign [4]. Although the motor fibers innervate the peroneal muscles, which are responsible for eversion and mild plantar flexion of the foot, any compression at the lower third of the leg does not cause any motor disturbances [8].

Diagnosis of SPN entrapment relies on clinical assessment supported by neurophysiological and imaging studies. Sensory nerve conduction tests and electromyography may reveal abnormalities such as prolonged latency or reduced sensory nerve action potentials, though results can appear normal due to anatomical variability. Imaging modalities, particularly MRI and ultrasound, are essential tools: MRI can show nerve swelling and increased T2 signal at the entrapment site, while ultrasound allows dynamic visualization of the SPN’s course and its emergence through the crural fascia [9].

Although the typical anatomy of the SFN is well established, various anatomical variations have been reported in its pattern of fascial penetration. A meta-analysis of 14 studies (665 limbs) found the most common pattern was SFN piercing the fascia as a single trunk, then bifurcating into MDN and IDN, with a prevalence of 82.7%. The Type 2 variant, involving early bifurcation before fascial penetration, occurred in 15.6% of cases, while Type 3, with absent IDN, was seen in 1.8% of limbs [10]. The present case aligns with the most common anatomical pattern (Type 1). However, it is clinically significant due to the unusual proximity of the nerve entrapment site to the lateral malleolus, a feature that may have implications for both diagnosis and surgical intervention.

Commonly, SPN entrapment is found 5-10 cm proximally to the lateral malleolus, as it is described by some studies [11,12]. Kuo and Wei and Akisue et al. found the entrapment site 5 cm above the lateral malleolus [13,14]. Johnston and Howell identified the entrapment site as being in proximity to the joint, which may be interpreted as within 5 cm of the lateral malleolus; however, the anatomical localization remains imprecisely defined in their description [15]. Similarly, Kernohan et al. presented entrapment above the lateral malleolus, which is not clearly defined [12]. The entrapment site has been characterized anatomically concerning the level of the ankle joint [16,17], the superficial exit point through the crural fascia, the level of the calf [18,19], and the topographical relationships with surrounding muscular structures [20-24]. In our case, the SPN was found entrapped at the piercing site of the crural fascia, almost 4.7 cm from the tip of the lateral malleolus. This anatomical finding is of particular interest, as, to the best of our knowledge and based on the available literature, entrapment of the SPN at such proximity to the lateral malleolus is a rare occurrence and has been reported only infrequently.

Entrapment of the SPN occurs due to pressure applied to the SPN 4 to 5 cm above the ankle joint. The nerve is constricted by the ligament-like tissue or fascia or by a muscle that may also come through the same limited space as the nerve through the fascia. The constriction of the nerve due to muscles causes localized trauma or injury to the limb.

The location of the compression plays a significant role in the diagnosis of SPN entrapment syndrome and decision-making regarding treatment. Treatment involves conservative and surgical measures. At the initial stages, include adjustments in lifestyle and alterations in activities that precipitate neuropathy [25]. If the symptoms persist, it is indicated that physiotherapy, corticosteroid injection, non-steroidal anti-inflammatory drugs, appropriate footwear, and rehabilitation for ankle instability [25]. Studies show that most patients needing surgery for SPN entrapment have previously undergone surgery to treat common peroneal nerve compression [26]. Surgical decompression or neurolysis is necessary in patients who were not permanently relieved with conservative treatment. Surgical decompression under open surgical or minimally invasive surgical intervention for decompression of the nerve is the choice of treatment if we have persistence of the symptoms [26, 27].

## Conclusions

SPN entrapment is a rarely recognized clinical entity that usually presents sensory alterations of the SPN distribution area over the anterolateral aspect of the lower leg and dorsum of the foot. Nerve compression has been described in the SPN as it exits from crural fascia in several sites, commonly 5-10 cm from the lateral malleolus. We present a cadaveric case where the SPN was found entrapped proximal to the crural fascia, 4.7 cm from the lateral malleolus. Awareness of possible nerve entrapment near the lateral malleolus is important for prompt and accurate diagnosis, enabling the development of an optimal treatment plan.
